# Detection and quantification by molecular techniques of early infection by *Lawsonia intracellularis* in suckling piglets

**DOI:** 10.1186/s40813-024-00394-6

**Published:** 2024-10-08

**Authors:** Víctor Rodriguez-Vega, Héctor Puente, Ana Carvajal, Lucía Pérez-Pérez, Samuel Gómez-Martínez, Fernando L. Leite, Rocío García, Lola Abella, Héctor Argüello

**Affiliations:** 1 Boehringer Ingelheim Animal Health España, Barcelona, Spain; 2 Departamento de Sanidad Animal, Facultad de Veterinaria, Universidad de León, León, Spain; 3 INDEGSAL, Universidad de León, León, Spain; 4 Boehringer Ingelheim Animal Health USA Inc, Duluth, GA USA; 5 Cárnicas Tabladillo, Segovia, Spain

**Keywords:** *Swine*, *Enteric infection*, *Transmission*, *Pathogen*, *Porcine proliferative enteropathy*, *Ileitis*

## Abstract

**Background:**

*Lawsonia intracellularis* is the causative agent of Porcine Proliferative Enteropathy (PPE), one of the most prevalent pig enteric diseases worldwide, but with sparse information about early infections in suckling piglets in the epidemiology of PPE. With that aim, this study evaluates the prevalence of *L. intracellularis* in 3-week-old piglets by analysing ileal digesta content and mucosal scrapings from 383 pigs from 16 farms (aprox., 25 pigs/batch) by real-time qPCR and droplet digital PCR (ddPCR).

**Results:**

Forty-nine samples yielded a qPCR positive result. Eleven samples from eight farms were confirmed as positive with concentrations of *L. intracellularis* from 3.5 log_10_ to 4.5 log_10_ bacteria/g of sample. Another 16 samples, eight farms, were classified as low positive (2.07–2.38 log_10_ bacteria/g) and 22 provided an uncertain result. Finally, 334 samples tested negative for *L. intracellularis*. At batch level, half of the farms included in the study had at least one positive sample and in 10 farms (62.5%) there was at least one low positive sample. The ddPCR was run in 50 of the 383 samples based on their PCR output (including low positive, uncertain and negative samples). Correlation analyses revealed a strong association between qPCR and the ddPCR results (ρ = 0.75; *p* < 0.001). The ddPCR allowed us to detect and confirm a positive result in the 19 samples classified as uncertain by the qPCR and detect *L. intracellularis* in 8 of 15 negatives by qPCR.

**Conclusions:**

The results of the study demonstrate that a number of piglets are already infected with *L. intracellularis* during the suckling period evidencing early infection in certain animals, adding information of PPE epidemiology and opening new research topics such as sow-piglet transmission. Study results also evidence the usefulness of a combination of qPCR and ddPCR to improve qPCR sensitivity but assuring high specificity.

## Background

*Lawsonia intracellularis* is an obligated intracellular bacterium taxonomically included in the familia Desulfovibrionaceae, which includes sulphate-reducing bacteria. It is a well-known pathogen of swine causing the Porcine Proliferative Enteropathy (PPE), one of the most prevalent pig enteric diseases worldwide [1]. The disease was first described in pigs in 1931 but prior to the intensification of pig production in the 1970´s, PPE was confined to occasional cases with acute or chronic disease outcomes. The true nature of PPE was discovered in 1973 when Alan Rowland and Gordon Lawson first saw the curved intracellular bacteria in Edinburgh, Scotland [2]. It would take another 20 years before *L. intracellularis* was cultured, identified and the Koch´s postulates determining the cause of PPE were fulfilled. The infection of undifferentiated epithelial cells in intestinal crypts causes abnormal proliferation of these cells and thickening of the intestinal epithelium more frequently in the ileum but may occasionally expand to the cecum and the colon regions [3]. *Lawsonia intracellularis* is considered an holoendemic pathogen, present on most of the commercial farms as was confirmed by a recent study which demonstrated direct detection by PCR in 90.3% of 144 herds and antibodies in 91.7% of 60 herds in Europe [4]. These results seem quite steady since a serological study performed in Spain [5] two decades ago revealed that *L. intracellularis* serum antibodies were found in 98% of the herds with the highest intra-herd prevalence in gilts and sows (83% and 88% respectively) [5]. Data worldwide supports European studies with similar Figs. [6–10]. Additionally, *L. intracellularis* was detected in the 93,6% of farms in China with a positive rate of 37,3% at pig level [6]. Serum antibodies were found in 100% of herds tested in Korea (the infection rate in individual pigs varied from 44 to 69%) [9], in 90.4% of 174 farms checked in USA, with intra-herd prevalence values ranging from 0.74–39.9% [10]. Furthermore, many infections are sub-clinicals [11, 12] and might not be chosen for diagnostic tests, a fact that implies underdiagnosis of this disease. Besides the clinical disease, PPE impacts on farm productivity and the economic losses associated to the disease have been estimated in at least 1 to 3 US$·per animal [13].

The introduction of *L. intracellularis* into a herd is mostly associated to infected pigs. Once inside a farm, the persistence of the pathogen between farms may be associated mostly to poor cleaning and disinfection of the facilities and by mixing pigs of different farms or ages [14, 15]. In addition, breeders, for instance subclinical infected sows, can act as amplifiers of the disease [16]. Although this last assertation is not completely evidenced or clear, shedding of *L. intracellularis* by peri-parturient sows has been reported, thus exposing suckling piglets to this pathogen [4]. Unfortunately, the information about the prevalence of *L. intracellularis* in suckling piglets is sparse.

Roasted piglet (called “tostón” in the central region of Spain) is a delicatessen oven-cooked food consumed all over Spain. Among the requisites included in the protected geographical indication (PGI) to produce tostón are a diet based only in mother’s milk, prohibition to administer antimicrobial treatments or iron injections either in piglets or their mothers and slaughtering at approximately twenty-one days of life, weighing between 5.2 and 7.3 kg. Due to their small size and weight, “tostón” pigs are slaughtered for human consumption in specialized commercial abattoirs. These piglets are raised usually in small familiar farms in which most of their production is addressed to this production.

Since suckling piglets may act as a source of *L. intracellularis* for next production stages, the information on its prevalence at the end of lactation would be key for implementing preventive strategies on the farm. So, we took advantage of the chance of collect intestinal samples in a slaughterhouse specialized in processing “tostón” with the aim of investigating the prevalence of *L. intracellularis* infection in approx.3-week-old piglets intended for human consumption. The results may help us to understand the potential role of early *L. intracellularis* infections, providing new insights into the epidemiology of PPE within farms in order to develop and implement strategies that help us to control the disease.

## Methods

### Study design

The samples used in this study were obtained in a Spanish slaughterhouse processing exclusively “tostón” piglets. Only pig farms which were included in the PGI took part in the study.

The study aimed at sampling a total of 15 commercial farms in the province of Segovia, Spain which were randomly selected in three different visits to the slaughterhouse and including a batch of 25 piglets per farm. Farms for “tostón” production are small to moderate in size (200 to 1,500 sows) and breed Large White x Landrace pigs within an indoor site-one production system with standard biosecurity and management procedures.

### Sample collection

At the evisceration point, the digestive tract of the chosen animals was collected. The duodenum, the ileocecal valve and the rectum were sealed and the upper tract including the stomach removed. Then, each intestine was individually bagged in sterile plastic bags which were identified with the batch and individual number. Intestines were transported to the laboratory under cooling conditions and processed immediately after arrival.

Once in the laboratory, the ileum from each animal was identified and a section of 5 to 10 cm was cut from the ileocecal valve upstream. The ileum digesta was squeezed into 1.5 mL Eppendorf tubes. When no digesta was available, the ileum segment was opened, the mucosa exposed and scraped without excessive pressure on the surface using a sterile glass slide. The obtained mucosa was placed into 1.5 mL Eppendorf tubes. From batch 8 to batch 16, we recorded if digesta, mucosa or a mix of digesta and mucosa were collected from each animal. All procedures were performed with strict measures of asepsia, avoiding microbial or DNA cross-contamination in the lab procedures.

### DNA extraction

DNA extraction from 200 mg of each sample was performed using GeneMATRIX™ Stool DNA kit (EurX™, Gdansk, Poland), following the manufacturer’s instructions. DNA samples were eluted in a volume of 100 µL and the concentration and purity of the DNA was measured using the spectrophotometer Nanodrop™ (Thermofisher Scientific). Final DNA concentration was diluted 1:10 in elution buffer for further PCR analyses. Samples were stored at -80° C until processing.

### Lawsonia intracellularis *real-time qPCR detection and quantification*

Each DNA sample was analysed in duplicate for *L. intracellularis* detection and quantification using a TaqMan qPCR as previously described [17]. Each reaction mixture (20 µL final volume) contained 8 µL of Maxima Probe real-time PCR Master Mix 2X (Thermo Scientific), 0.3 µL of 10 µM of each primer, 0.2 µL of 10 µM Taq-Man probe, 0.12 µL of Rox (diluted 1:10 in nuclease-free water, Thermo Scientific), 2 µL of the template DNA and nuclease-free water up to 20 µL. The qPRCs were run in a QuantStudio 1 thermal cycler (Applied Biosystems). The concentration of *L. intracellularis* in each sample was estimated using an in-house standard curve which was prepared by ten-fold serial dilutions of a *L. intracellularis* preparation with approximately 5 log_10_*L.* intracellularis/mL (range 5 log_10_ to 0 log_10_ bacteria/mL). Results were finally expressed by the number of *L. intracellularis*/g of intestinal content.

Results were interpreted as follows; samples with a concentration ≥ 3 log_10_*L. intracellularis*/g of digesta/mucosa were considered positive while samples in the range between 3 log_10_ (1000) and 2.2 log_10_ (200) were considered low-concentration positives. Concentrations between 2.2 log_10_ and 1.8 log_10_ (180) were classified as uncertain results, defined as data with amplification curves which could not be clearly classified as positive, but which exhibited certain amplification. Finally, amplifications below 1.8 log_10_*L. intracellularis*/g of digesta/mucosa and samples with no amplification were considered negative. Amplification curves were carefully checked, and digital PCRs were performed to detect and quantify *L. intracellularis* DNA.

### Droplet Digital PCR protocol(ddPCR)

A ddPCR method was set up to double check the diagnosis in a selection of low positive, uncertain and negative samples by qPCR. These samples (50 in total) were selected base on their qPCR result, trying to balance numbers in each qPCR category.

*Lawsonia intracellularis* detection and quantification by ddPCR was performed using the Quantstudio Absolute Q System (Thermofisher Sciencitific™) according to the manufacturer instructions. Each sample was evaluated in duplicate in a reaction mix with 2 µL of target DNA and 9 µL of reaction volume. Data was analysed with the corporative software Quantstudio Absolute (Thermofisher Sciencitific™) to estimate the total number of droplets measured and the number of positive droplets in each sample.

For ddPCR analyses the limit of detection (LOD) and limit of blank (LOB) parameters were estimated according to previous references [18] The LOD was assessed by estimating ddPCR counts in three samples with a known *L. intracellularis* concentration estimated by qPCR. DNA from these samples was 1:10 diluted down to 0.1 log_10_ and performing the ddPCR in triplicate. The LOB was defined as the highest number of *L. intracellularis* copies detected in 12 blank samples. By the information provided by both parameters, the cut-off value for the ddPCR-*L. intracellularis* assay was set at 7 droplets in 9 µL of reaction volume.

### Statistical analyses

Data was recorded in an Excel spreadsheet (Microsoft Office©) and further analysed using either Excel or R version 4.2.1 (R core team 2021). Potential differences in distribution of positive samples among sampling days, farms or sort of sample collected was analysed by Chi^2^ or Fisher’s exact tests for categorical variables and an ANOVA test for ddPCR counts in the three categories of qPCR (low positive, uncertain and negative) analysed. Correlations among qPCR concentrations and the number of positive droplets in ddPCR were performed using a Person correlation test after confirming data normality. Figures were prepared either in Excel or using the *ggplot2* (version 3.4.3) package in R.

## Results

Data from the sixteen farms finally included in the study are detailed in Table 1. All farms involved a total of 25 animals sampled except batch 11 with 24 samples, batch 16 with 22 samples and batch 2 where only 12 pigs could be sampled. Samples from the first seven farms were collected in sampling 1, batch 8 to batch 13 were processed in the second sampling and the last three farms were collected in the last visit (sampling 3). These figures provided a final number of 383 samples. In 200 of these samples (from batch 8 to batch 16), we recorded if DNA was obtained from the digesta content, from the ileum mucosa or a combination of both. Thus, a total of 89 DNA samples were processed from digesta, 67 from ileum mucosa scrapings and the other 44 from a mix of digesta and ileum mucosa.

**Table 1:** Summary of the data and results from the 15 farms included in the study to analyse the early transmission of *Lawsonia intracellularis*

Farm	Sampling	Date	No samples processed	PCR results			
				Positive^1^	Low positive^1^	Uncertain^1^	Negative
Farm 1	Sampling 1	17/03/2022	25	1	2	2	20
Farm 2	Sampling 1	17/03/2022	12	0	0	0	12
Farm 3	Sampling 1	17/03/2022	25	0	0	1	24
Farm 4	Sampling 1	17/03/2022	25	2	2	2	19
Farm 5	Sampling 1	17/03/2022	25	0	0	0	25
Farm 6	Sampling 1	17/03/2022	25	0	0	0	25
Farm 7	Sampling 1	17/03/2022	25	0	0	0	25
Farm 8	Sampling 2	16/05/2022	25	1	2	5	17
Farm 9	Sampling 2	16/05/2022	25	1	2	1	21
Farm 10	Sampling 2	16/05/2022	25	1	1	1	22
Farm 11	Sampling 2	16/05/2022	24	1	1	0	22
Farm 12	Sampling 2	16/05/2022	25	1	2	1	21
Farm 13	Sampling 2	16/05/2022	25	3	2	4	16
Farm 14	Sampling 3	05/07/2022	25	0	1	0	24
Farm 15	Sampling 3	05/07/2022	25	0	0	2	23
Farm 16	Sampling 3	05/07/2022	22	0	1	3	18

^1^ qPCR interpretation: samples with a concentration ≥ 3 log_10_ intracellularis/mg of digesta/mucosa was considered positive while samples in the range between concentrations of 2.2 log_10_ and 3 log_10_ were considered low-concentration positives and concentrations between 2.2 log_10_ and 1.8 log_10_ uncertain results

### Confirmed positive samples by qPCR

Forty-nine samples yielded a positive result with amplification curves compatible with the presence of *L. intracellularis* DNA. Among them, eleven samples (2.87%; 95% CI [1.21-4.53%]) were confirmed as positive with concentrations of *Lawsonia* ranging from 3.5 log_10_ bacteria/gram of sample to 4.5 log_10_ bacteria/gram of sample. Ct values of these samples ranged between 25.3 and 29.6.

These positive animals belonged to eight different farms and were distributed among the three samplings performed. Four of the positive samples were obtained from digesta, another two from mucosa samples and other two from a mix of digesta and mucosa. No significant differences were observed among samplings (*p* = 0.17) or type of sample (*p* = 0.12).

Another 16 samples were classified as low positive samples (4.18%; 95% CI [2.19-6.17%]). The concentrations estimated in these samples varied from 2.07 log_10_ bacteria /gram of sample and 2.38 log_10_ bacteria/gram of sample. Ct values of these samples ranged between 29.5 and 31.5 in this group of samples. These low positive samples were linked to eight farms, also in the three samplings (*p* = 0.19) and with no significant differences in positives among digesta, mucosa or a mix of digesta and mucosa (*p* = 0.25).

A larger number of samples, 22 in total (5.74%; 95% CI [3.41-8.06%]), provided an uncertain result. Concentration values in these samples varied from 1.9 log_10_ bacteria /gram to 2.16 log_10_ bacteria /gram of sample with Ct values from 31.1 to 32.5. Ten out of the sixteen farms had at least a sample with uncertain diagnostic result. Eight were from digesta, another four from mucosa and two from mixed samples. Again, no differences were found by sampling (*p* = 0.09) or sample type (*p* = 0.32).

Finally, 334 samples tested negative for *L. intracellularis* (87.21%; 95% CI [83.89-90.53%]). From the negative samples, 276 had a signal which provided an amplification result while another 58 did not show any amplification signal, thus they were classified as negative.

### Results by pig farm

**Fig. 1:**
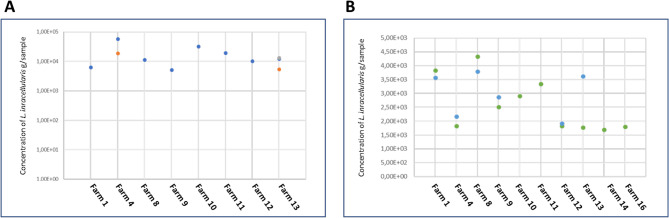
Concentration of *Lawsonia intracellularis* estimated by gram of sample (digesta and/or ileum mucosa) in positive piglets from the 15 farms tested. Figure 1**A** shows results from positive samples in eight farms and Fig. 1**B** shows summarizes the data from low positive samples detected in piglets from 12 farms

At farm level, half of the farms included in the study had at least one positive sample and in 10 of them there was at least one low positive sample (62.5%) (Fig. 1). Interestingly there was a good alignment of positive samples and low positive samples; all farms with a positive had at least a low positive and only two farms with low positives had no positive samples (Table 1).

Just in four farms (25%) all samples were negative, that means without positive, low positive or uncertain sample results. Except in samples from farm 13 and farm 4 with three and two positive samples respectively, the other six positive farms only had a positive sample (Table 1). The number of samples with a low positive result was not higher in the positive farms, it just ranged from 1 to 2 samples per batch. Altogether, considering a mean number of 25 pigs analysed per farm, the median prevalence of positive pigs per farm was 4% of the pigs tested (one pig per farm) with a maximum of 12% (three positive pigs detected in a single farm). Similarly, looking at low positive results data, the median prevalence of the pigs tested per batch was 8%, which means two low positive samples in positive farms.

### Droplet digital PCR results

Quantification of *L. intracellularis* by ddPCR was performed in 50 of the 383 samples. We selected these 50 samples based on their PCR output. We included a qPCR positive sample, 15 samples categorized as “low positive”, 19 samples with an “uncertain” qPCR result and 15 samples that tested negative by qPCR but with amplification signals. Thus, ddPCR analyses were performed to determine with higher accuracy the presence of *L. intracellularis* in samples categorized by qPCR as “low positive”, “uncertain”, and “negative”.

First, we set up the method for first time in our laboratory. Once it was confirmed that the qPCR conditions were able to adequately amplified DNA in the ddPCR, enabling to run the method with confidence, parallel experiments were run to estimate the LOB and LOD values and define a cut-off which could be reliable in specificity for the ddPCR method. The limit of detection was set at 7 droplets in the final volume tested (9 µL) by the results provided by blank samples, negative controls and diluted 1:10 positive samples (data not shown). Anything below that cut-off was considered as negative.

**Fig. 2:**
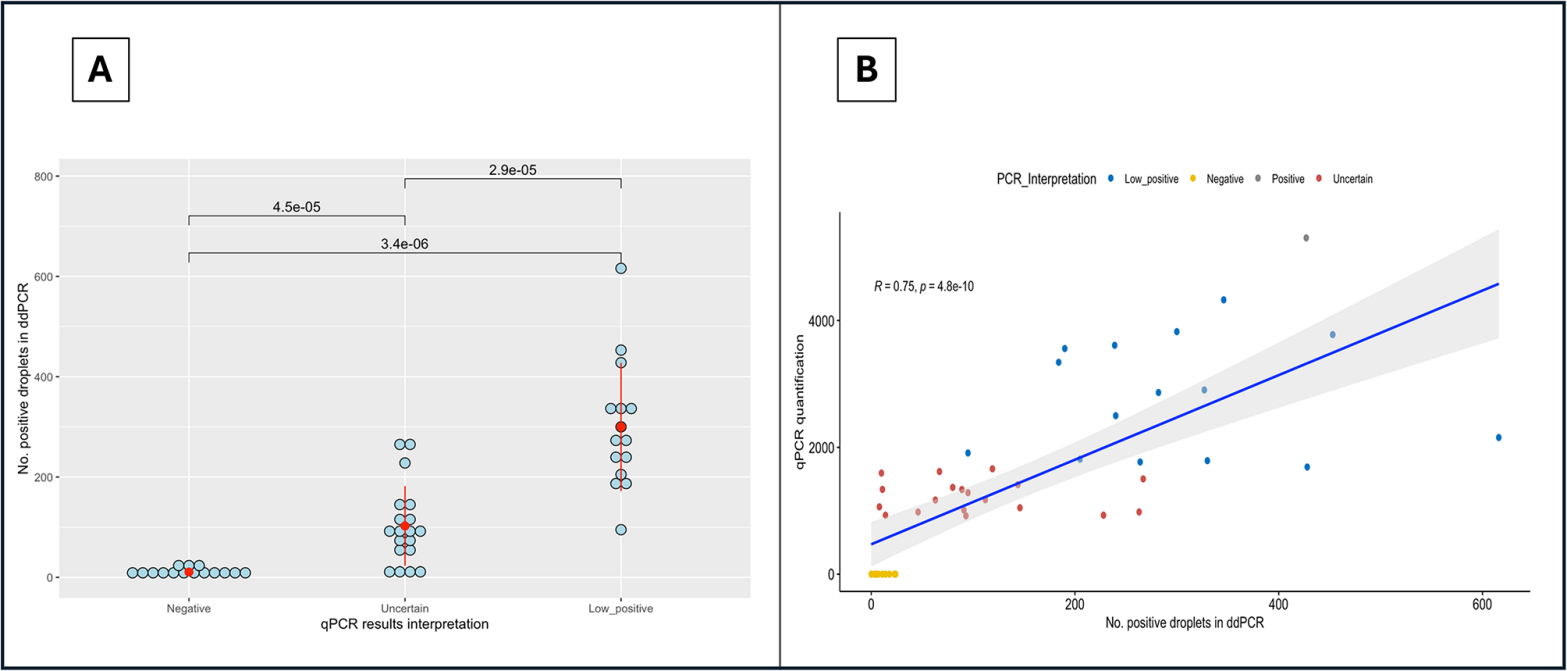
*Lawsonia intracellularis* droplet digital PCR results obtained in 50 samples collected from piglets. Figure 2**A** droplet counts in samples categorized as negative, uncertain or low positive by qPCR. Whiskers show significant differences among categories. Figure 2**B** correlation analysis between qPCR quantification and droplet counts. Pearson correlation ^®^ and P-value estimated for the data analysed

The results of the ddPCR (droplet counts estimated in 9 µL) ranged from 0 in a negative qPCR sample to 616 in a sample categorized as “low positive” by qPCR. It was observed significant differences in the number of positive droplets detected (*p* < 0.001) between qPCR output categories (“low positive”, “uncertain”, and “negative”) (Fig. 2A; Table 2). Similarly, correlation analyses revealed a strong association between the estimated quantifications by qPCR and the ddPCR results (ρ = 0.75; *p* < 0.001; Fig. 2B) and also significant association but with lower association between qPCR Ct value and ddPCR counts (ρ =- 0.63; *p* < 0.001).

**Table 2:** *Lawsonia intracellularis* detection and quantification by droplet digital PCR in a selection of 50 DNA samples previously analysed by qPCR

qPCR result^1^	No. samples		Droplet digital PCR result		
			Mean number droplets detected (max-min)	No. (+) samples	No. (-) samples
Low positive	15		269 (616-3)	14	1
Uncertain	19		102 (267-8)	19	0
Negative	15		10.5 (24 − 3)	8	7

^1^By qPCR concentration samples were classified as low positive (3 log_10_ to 2.2 log_10_), uncertain (2.2 log_10_) and negative (< 1.8 log_10_)

All low-concentration positive samples except one were positive by ddPCR with droplet counts from 3 (negative sample) to 616 droplets. Interestingly, the ddPCR allowed to detect and confirm a positive result in the 19 samples classified as “uncertain” by the qPCR. Additionally, 8 samples from the fifteen evaluated and defined as “negative” by the qPCR interpretation, tested positive using the ddPCR method. Droplet counts in these 8 samples ranged from 8 positive droplets to 24 positive droplets.

## Discussion

*Lawsonia intracellularis* is a successful intestinal pathogen which is currently present on most of the commercial pig farms worldwide [3, 4, 6]. The large on farm faecal prevalence and seroprevalence observed in growing and finishing stages respectively [1, 4] may be linked to infections occurring at these two stages but also could be initiated earlier on, during lactation period, when some piglets may become infected by exposure to their mother feces. The information of prevalence of *L. intracellularis* at the end of suckling period is of relevance to better understand the epidemiology of the infection in pig production and revise current preventive strategies in *L. intracellularis* control.

With that aim, here we have performed to the best of our knowledge, the first study in which the potential infection in sucking piglets is evaluated in the intestinal section targeted by *L. intracellularis*, the ileum. Toston production offered the chance to collect over 380 intestinal samples from around three-week-old piglets for “tostón” carcass production. It is important to mention that “tostón” production exhibits certain particularities, such as lack of iron injection or lack of antimicrobial treatments. In particular antimicrobial treatments may impact *L. intracellularis* prevalence figures, hence this particular aspect needs to be considered when extrapolating our data. It is also remarkable that within the EU the use of antimicrobials is suffering a drastic reduction and prophylactic treatments are already banned [19], thus the scenario proposed in our research probably resembles the reality of many herds.

The sample size for this study was calculated for a herd prevalence relatively high, of at least 12% of qPCR positive samples, considering both the age of piglets and the production stage. Although a larger sample size could fine-tune the prevalence estimation, that the results obtained in this explanatory study are of sufficient relevance. Indeed, from the 16 farms included in the three visits to the slaughterhouse, 75% had at least one positive animal and in half of the farms analysed, it was observed pathogen concentrations of relevance in the intestine (up to 4.5 log_10_ g/ sample). In this sense, none of the pigs tested exhibited concentrations associated by previous studies to clinical disease (approx., 6 log_10_ g/feces; [12, 20–22]) but the eight samples classified as positive were in concentrations above the 3.3 log_10_ g/sample estimated in the same study for non-pathological shedding. Thus, these animals could already be under an active process which may end in gross lesions weeks after and be source of infection for other piglets.

The presence of maternally derived antibodies has been described at this age [5, 23, 24] and it is generally assumed that they protect against *L. intracellularis* infection during the lactation period, so the onset of the infection occurs a few weeks after weaning, when this maternal immunity vanishes [3]. However, it is also demonstrated that do not always prevent pigs from becoming infected [25]. Considering all positive qPCR results, the study prevalence was 12.8%, a value similar to the figures obtained in a recent study in China that showed an 8.9% of qPCR positive results from fecal samples from weaners [6]. At batch level, the number of positive or low positive samples was below 15% in most of the farms, thus we can infer that the number of pigs in which *L. intracellularis* is replicating actively in the gut is quite low, despite most of the farms have a few of these infected animals. These figures contrast to those reported in recent literature about prevalence values in subsequent production stages, growing and finishing, on European countries (17.7% among nursery pigs, 33.0% among growing pigs and 27.8% among finishing pigs) [4] and on Chinese farms (93.6%) [6]. The increase of prevalence has been demonstrated as well, by seroprevalence studies that show that the number of seropositive animals increases from 10% at the end of nursery to 67% at the end of fattening [23]. Our result allows to hypothesize that the prevalence and shedding peaks observed in later stages may be in part related to fecal-oral infection from these early-seeders to susceptible pigs, at least until the majority of the population is protected. It is now time to decipher if that transmission involves the sow, the farrowing environment or other aspects such as wild animals or reservoirs such as rodents [26].

The droplet digital PCR (ddPCR) is an emerging PCR assay, based on water-oil emulsion droplet technology that has been reported to be more sensitive and accurate than qPCR for the diagnosis of several infectious pathogens, especially in the case of low-copy nucleic acids [27–30]. We set up this method for first time in *L. intracellularis* detection by using a DNA target al.ready described for qPCR elsewhere [17]. Interestingly, ddPCR offered a good correlation with qPCR results validating its accuracy. The higher the concentration of *L. intracellularis*, the better the correlation with ddPCR results. Besides, we observed significant differences in ddPCR positive droplet counts among the three categories of qPCR positives established, positive, low positive and uncertain. This result helped to confirm that the arbitrary categories established by the extrapolated quantities obtained in the reference curve in the qPCR. As already mentioned, ddPCR offers the possibility to fine-tune DNA concentration in certain samples and may improve the sensitivity of other techniques [28, 30]. In our study 22 samples provided an uncertain result based on their large Ct-value and low intensity amplification curve observed in the qPCR. Large Ct values may be associated to low concentrations of the target DNA, or instead off-target products or artifacts such as primer-dimers [31]. We performed a ddPCR analysis in 19 samples with an uncertain result in qPCR and 15 samples with negative result in qPCR. All uncertain samples were confirmed as positive but also half of the negative samples tested had ddPCR counts over the limit of detection, thus confirming the presence of *L. intracellularis* DNA, but in almost negligible concentration. Thus, the results evidence the usefulness of a combination of qPCR and ddPCR to improve qPCR sensitivity but assuring high specificity, already demonstrated by previous studies with different infectious pathogens [27, 28, 30]. Undoubtedly, the double technique approach seems of interest within a low pathogen load context such as the study described here.

## Conclusion

Altogether the results of the study demonstrate that a number of piglets already are infected with *L. intracellularis* during the lactation period. While the demonstrated prevalence in this study was low, it opens a new research window to decipher the role of these early infected animals in the infections observed at the post-weaning period. In addition, the study underlines the potential role of sow-piglet transmission in the epidemiology of *L. intracellularis* which may be added to piglet to piglet transmission and environment or wild animals (rodents mostly) reservoirs. This is crucial for developing and implementing preventive measures and methods that help us control the disease, especially in multi-site system, where all-in all-out and thoughtful cleaning protocols limit environmental contamination. Undoubtedly, both the impact of early infection, not only in pigs with relevant concentration of *L. intracellularis* in their intestine but also with low counts and firewalls to mitigate the vertical transmission are topics of interest for future studies. Beside, the prevalence at weaning should also be considered when developing and implementing vaccination strategies aimed to reduce the economic impact of proliferative enteropathy in growing and finishing pigs.

## Data Availability

No datasets were generated or analysed during the current study.

## Declarations

### Authors’ information

Lucía Pérez-Pérez (PRE2020-093762) is supported by the Spanish Ministry of Education. Samuel Gómez-Martínez is granted by the Junta de Castilla y Leon pre-doctoral programme (ORDEN EDU/1868/2022).

## Abbreviations

PPE: Porcine Proliferative Enteropathy
USA: United States of America
US$: United States dollar
PGI: protected geographical indication
DNA: Desoxyribonucleic acid
PCR: polymerase chain reaction
ddPCR: Droplet Digital PCR protocol
qPRC: quantitative polymerase chain reaction
LOD: limit of detection
LOB: limit of blank. ANOVA: Analysis of variance
Ct: Cycle threshold
